# Cytokeratin 5/6 fingerprinting in HER2-positive tumors identifies a poor prognosis and trastuzumab-resistant Basal-HER2 subtype of breast cancer

**DOI:** 10.18632/oncotarget.3106

**Published:** 2015-01-29

**Authors:** Begoña Martin-Castillo, Eugeni Lopez-Bonet, Maria Buxó, Joan Dorca, Francesc Tuca-Rodríguez, Miguel Alonso Ruano, Ramon Colomer, Javier A. Menendez

**Affiliations:** ^1^ Unit of Clinical Research, Catalan Institute of Oncology, Girona, Catalonia, Spain; ^2^ Girona Biomedical Research Institute (IDIBGI), Molecular Oncology Group, Girona, Catalonia, Spain; ^3^ Department of Anatomical Pathology, Dr. Josep Trueta Hospital of Girona, Girona, Catalonia, Spain; ^4^ Epidemiology Unit and Cancer Registry of Girona (UERCG), Catalan Cancer Plan, Catalan Health Government, Girona, Catalonia, Spain; ^5^ Department of Nursing, Universitat de Girona (UdG), Girona, Catalonia, Spain; ^6^ Medical Oncology Department, Catalan Institute of Oncology, Girona, Catalonia, Spain; ^7^ Department of Gynecology, Dr. Josep Trueta Hospital of Girona, Girona, Catalonia, Spain; ^8^ Breast Cancer Clinical Research Unit, CNIO-Spanish National Cancer Research Center, Madrid, Spain; ^9^ Medical Oncology Department, Hospital La Princesa, Madrid, Spain; ^10^ Translational Research Laboratory, Catalan Institute of Oncology (ICO), Girona, Catalonia, Spain

**Keywords:** Breast cancer, HER2, basal-like, trastuzumab, cytokeratins

## Abstract

There is an urgent need to refine the prognostic taxonomy of HER2+ breast carcinomas and develop easy-to-use, clinic-based prediction algorithms to distinguish between good- and poor-responders to trastuzumab-based therapy. Building on earlier studies suggesting that HER2+ tumors enriched with molecular and morpho-immunohistochemical features classically ascribed to basal-like tumors are highly aggressive and refractory to trastuzumab, we investigated the prognostic and predictive value of the basal-HER2+ phenotype in HER2-overexpressing tumors. Our retrospective cohort study of a consecutive series of 152 HER2+ primary invasive ductal breast carcinomas first confirmed the existence of a distinct subgroup co-expressing HER2 protein and basal cytokeratin markers CK5/6, the so-called basal-HER2+ phenotype. Basal-HER2+ phenotype (≥10% of cells showing positive CK5/6 staining), but not estrogen receptor status, was significantly associated with inferior overall survival by univariate analysis and predicted worsened disease free survival after accounting for strong prognostic variables such as tumor size at diagnosis in stepwise multivariate analysis. In the sub-cohort of HER2+ patients treated with trastuzumab-based adjuvant/neoadjuvant therapy, basal-HER2+ phenotype was found to be the sole independent prognostic marker for a significantly inferior time to treatment failure in multivariate analysis. A CK5/6-based immunohistochemical fingerprint may provide a simple, rapid, and accurate method for re-classifying women diagnosed with HER2+ breast cancer in a manner that can improve prognosis and therapeutic planning in patients with clinically aggressive basal-HER2+ tumors who are not likely to benefit from trastuzumab-based therapy.

In women with early-stage breast cancer, adjuvant use of the anti-HER2 monoclonal antibody trastuzumab reduces recurrence risk when added to standard chemotherapy [1, 2]. However, not all cases of HER2+ breast tumors derive similar benefit from trastuzumab because a significant number of patients develop disease recurrence. At present, clinicians rely on established markers of HER2 expression for selecting patients for adjuvant trastuzumab or neoadjuvant pertuzumab-trastuzumab therapy i.e., immunohistochemical expression at the 3+ level or FISH ratio of 2 or greater [3]. Biomarkers to identify those patients who are not likely to benefit from trastuzumab would be clinically useful, allowing patients to pursue other therapeutic options. A predictive biomarker to classify a subgroup of patients with HER2+ tumors that are particularly resistant to trastuzumab could be especially important in the adjuvant setting, where the effectiveness of a given therapy in an individual patient cannot be assessed. Unfortunately, the identification of a robust clinical or molecular predictor of adjuvant trastuzumab benefit, including HER2 itself, has proven challenging [4–8].

Earlier work by Harris *et al*. [9], demonstrated that a particular HER2+ tumor phenotype overexpressing genes associated with the basal-like phenotype, including higher expression of basal cytokeratins (CKs), was more frequent in the non-responding group of patients receiving pre-operative trastuzumab than in the responding group. Building on these pioneering findings and considering that trastuzumab sensitivity is notably restricted to luminal-HER2+ breast cancer cell lines, whereas all basal-HER2+ cell lines exhibit inherent primary resistance to trastuzumab [10], we recently proposed that a basal CK surrogate definition of HER2+ breast carcinomas might define subgroups of patients likely to display resistance to trastuzumab-based therapy. Although published data on HER2+ and basal phenotype are limited, the longstanding assumption that HER2+ and basal-like breast cancers are mutually exclusive entities is open to dispute. While the first molecular portraits of breast tumors using DNA microarrays suggested that breast carcinomas with a basal phenotype are HER2 non-amplified [11], subsequent refined analyses tended to include HER2-amplified tumors branching close or included in a *bona fide* basal-like subclass [12–15]. Beyond microarray-based gene profiling studies, the so-called basal-HER2+ subtype has been also identified by immunohistochemical biomarker profiles. When Laakso *et al*. segregated basal-like breast cancers based on immunohistochemical expression of basal CKs, those tumors with low basal CK expression were likely to have HER2 overexpression [16]. Similarly, Liu *et al*. described a small group of hormone receptor-negative tumors simultaneously expressing HER2 and basal markers [17]. Both studies found that patients with the basal-HER2+ subtype had a significantly worse prognosis than those with basal-like and HER2+ tumors. Bhargava *et al*. [18] and our own group [19] observed that many morphological and immunohistochemical features classically ascribed to basal-like tumors, including large geographic necrosis and lymphoid infiltrate, are commonly seen in basal-HER2+ tumors. Bagaria *et al*. [20] have recently confirmed that when luminal-HER2+ (ER-positive and basal CK-negative), HER2+ (ER-negative and basal CK-negative) and basal-HER2+ (ER-negative and basal CK-positive) were correlated with clinicopathological features and overall survival, the basal-HER2+ subtype was associated with the worst prognosis.

Collectively, the results from these studies strongly support the notion that the basal-HER2+ phenotype may delineate a distinct entity of biologically-aggressive breast carcinomas; however, whether the basal-HER2+ phenotype also has clinical utility as a predictive marker of resistance to trastuzumab-based therapy remains to be clarified. Beyond confirming that the basal-HER2+ phenotype can predict worse disease-free and overall survival, we have also evaluated whether immunohistochemical-based identification of the basal-HER2+ phenotype can predict resistance to trastuzumab-based adjuvant therapy, which may have crucial implications for patients originally identified as suitable for trastuzumab based solely on their HER2+ phenotype. We show here that a simple CK5/6-based fingerprint using a 10% positivity cutoff, allows the re-classification of HER2+ tumors in a manner that improves prognosis and therapeutic planning in a sub-class of patients with clinically aggressive basal-HER2+ tumors that are not likely to benefit from trastuzumab-based therapy.

## RESULTS

### Clinicopathological features

**Figure 1:**
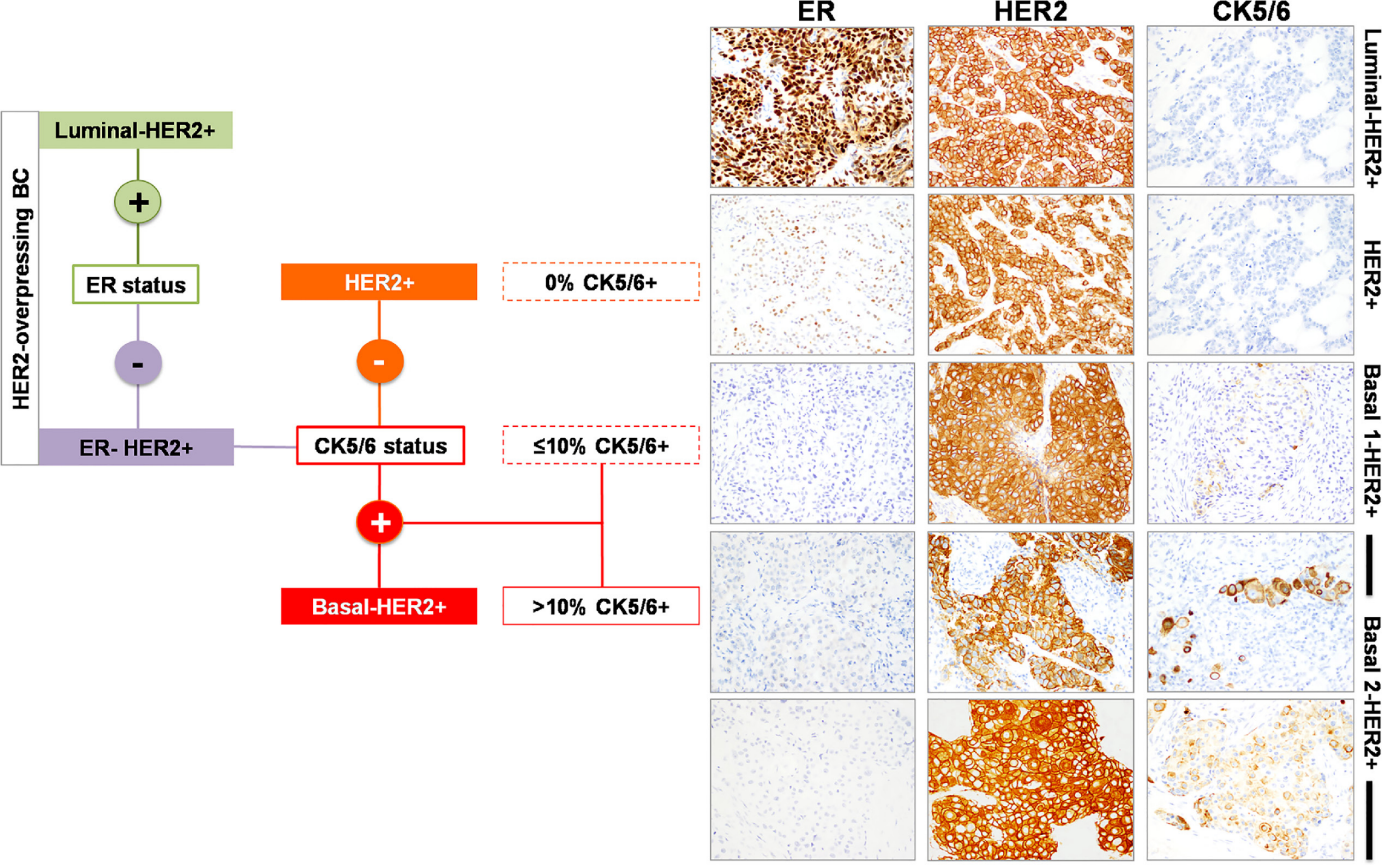
Immunophenotypic classification of HER2-overexpressing breast carcinomas

Breast cancer tissue sections of 154 consecutive patients with HER2-overexpressing primary invasive ductal breast cancer were evaluated for expression of ER and CK5/6 by IHC: 89 (58%) tumors were luminal-HER2+, 39 (26%) tumors were HER2+, and 24 (16%) tumors were basal-HER+ (Table 1). Representative examples of HER2+ breast carcinomas expressing basal epithelial CK markers (CK5/6) are shown in Figure 1.

**Table 1:** Patient and tumor characteristics

Characteristic	Luminal-HER2+	Basal-HER2+	HER2+	Basal-HER2+ vs others	Luminal-HER2+ vs HER2+
	*n* (%)	*n* (%)	*n* (%)	*P* value^*a^	*P* value^*a^
No. of patients	89 (58.6)	24 (15.8)	39 (25.7)		
Age, years, mean ± SD	58.2 ± 16.4	63.1 ± 15.3	56.67 ± 14.2	0.130^*1^	0.591^*1^
Tumor size					
T1	34 (38.2)	3 (12.5)	13 (33.3)	0.011 / 0.008^*2^	0.350 / 0.345^*2^
T2	41 (46.1)	10 (41.7)	17 (43.6)		
T3+T4	6 (6.7)	8 (33.3)	7 (17.9)		
Inflammatory	5 (5.6)	2 (8.3)	2 (5.1)		
Unknown	3 (3.4)	1 (4.2)	0 (0.0)		
Node status					
Negative	42 (47.2)	7 (29.2)	19 (48.7)	0.177 / 0.097^*2^	0.566 / 0.943^*2^
Positive	43 (48.3)	16 (66.7)	20 (51.3)		
Unknown	4 (4.5)	1 (4.2)	0 (0.0)		
Tumor grade					
1	3 (3.4)	0 (0.0)	1 (2.6)	0.934 / 0.892^*2^	0.435 / 0.478
2	25 (28.1)	5 (20.8)	8 (20.5)		
3	43 (48.3)	14 (58.3)	25 (64.1)		
Unknown	18 (20.2)	5 (20.8)	5 (12.8)		
Adjuvant trastuzumab					
No	40 (44.9)	13 (54.2)	20 (51.3)	0.514 / 0.738^*2^^*b^	0.793 / 0.594^*2^^*b^
Yes	42 (47.2)	11 (45.8)	17 (43.6)		
Unknown	7 (7.9)	0 (0.0)	0 (0.0)		
Adjuvant chemotherapy					
No	21 (23.6)	4 (16.7)	8 (20.5)	0.143 / 0.394^*2^^*b^	0.878 / 0.647^*2^^*b^
Yes	57 (64.0)	20 (83.3)	27 (69.2)		
Unknown	11 (12.4)	0 (0.0)	4 (10.3)		
Adjuvant hormone therapy					
No	7 (7.9)	21 (87.5)	37 (94.4)	<0.001 / <0.001^*2^^*b^	<0.001 / <0.001^*2^^*b^
Yes	71 (79.8)	3 (12.5)	2 (5.1)		
Unknown	11 (12.4)	0 (0.0)	0 (0.0)		
First clinically relevant event					
Local recurrence	1 (1.1)	2 (8.3%)	1 (2.6)	0.007 / 0.004^*2^	0.699 / 0.861^*2^
Distant metastasis	11 (12.4)	7 (29.2)	7 (18.4)		
Contralateral breast cancer	0 (0.0)	2 (8.3)	0 (0.0)		
Second primary tumor	5 (5.6)	1 (4.2)	2 (5.3)		
Not occurred	58 (65.2)	9 (37.5)	24 (63.2)		
Unknown	8 (9.0)	1 (4.2)	1 (2.6)		
Death from other causes	6 (6.7)	2 (8.3)	3 (7.9)		
First metastases site					
Not occured	79 (88.8)	17 (70.8)	33 (84.6)	0.084	0.773
Visceral	3 (3.4)	2 (8.3)	1 (2.6)		
No visceral	4 (4.5)	2 (8.3)	3 (7.7)		
Both	3 (3.4)	3 (12.5)	2 (5.1)		

*1 Parametric test: Independent two-sample Student's *t* test

*2 excludes unknown category

*a Fisher's exact test

*b Chi-square test

Patients with basal-HER2+ tumors were more likely to have larger tumors (*P* = 0.011) and recurrence (*P* = 0.007) than those with luminal-HER2+ and HER2+ tumors. There were no differences in age at diagnosis, tumor size, nodal status, and tumor grade between patients with luminal-HER2+ and HER2+ tumors. There were no differences in the use of adjuvant trastuzumab and chemotherapy across the three HER2+ groups. Patients with luminal-HER2+ tumors were more likely to receive hormone therapy than patients with HER2+ and basal-HER2+ tumors (*P* < 0.001) (Table 1).

To exclude equivocal reactions, clinicopathological features were reassessed after stratification of the basal-HER2+ tumors (CK5/6 staining score > 0) to underlying CK5/6 expression pattern: basal 1-HER2+ (<10% of cells showing positive staining) and basal 2/HER2+ (≥10% of cells showing positive staining) (Table 2). When a positive CK5/6 staining in ≥ 10% of the HER2+ breast cancer tissue sections was registered as a diagnostically relevant positive reaction [21], patients with basal 2-HER2+ tumors (but not basal 1-HER2+ tumors) were older (*P* = 0.034) and more likely to have larger tumors (*P* = 0.003) and recurrence (*P* < 0.001) than those with luminal-HER2+ and HER2+ tumors. When compared to HER2+ tumors, patients with basal 2-HER2 tumors (but not basal 1-HER2+ tumors) were older (*P* = 0.035) more likely to have larger tumors (*P* = 0.022) and recurrence (*P* = 0.012) (Table 2).

**Table 2:** Patient and tumor characteristics (stratification by CK5/6 expression status)

Characteristic	Luminal-HER2+	Basal 1-HER2+	Basal 2-HER2+	HER2+	Basal 1-HER2+ vs others	Basal2-HER2+ vs others	Basal1-HER2+ vs HER2+	Basal2-HER+ vs HER2+
	*n* (%)	*n* (%)	*n* (%)	*n* (%)	*P* value^*a^	*P* value^*a^	*P* value^*a^	*P* value^*a^
No. of patients	89 (58.6)	12 (7.9)	12 (7.9)	39 (25.7)				
Age, years, mean ± SD	58.2 ± 16.4	57.83 ± 14.0	68.33 ± 15.3	56.67 ± 14.2	0.935^*1^	0.034^*1^	0.739^*1^	0.035^*1^
Tumor size								
T1	34 (38.2)	3 (25.0)	0 (0.0)	13 (33.3)	0.483 / 0.360^*2^	0.003 / 0.002^*2^	0.849 / 0.849^*2^	0.022 / 0.048^*2^
T2	41 (46.1)	5 (41.7)	5 (41.7)	17 (43.6)				
T3+T4	6 (6.7)	3 (25.0)	5 (41.7)	7 (17.9)				
Inflammatory	5 (5.6)	1 (8.3)	1 (8.3)	2 (5.1)				
Unknown	3 (3.4)	0 (0.0)	1 (8.3)	0 (0.0)				
Node status								
Negative	42 (47.2)	2 (16.7)	5 (41.7)	19 (48.7)	0.092 / 0.036^*2^	0.515 / 0.531^*2^	0.091 / 0.091^*2^	0.311 / 1.000^*2^
Positive	43 (48.3)	10 (83.3)	6 (50.0)	20 (51.3)				
Unknown	4 (4.5)	0 (0.0)	1 (8.3)	0 (0.0)				
Tumor grade								
1	3 (3.4)	0 (0.0)	0 (0.0)	1 (2.6)	0.847 / 1.000^*2^	0.656 / 1.000^*2^	1.000 / 1.000^*2^	0.453 / 1.000^*2^
2	25 (28.1)	3 (25.0)	2 (16.7)	8 (20.5)				
3	43 (48.3)	8 (66.7)	6 (50.6)	25 (64.1)				
Unknown	18 (20.2)	1 (8.3)	4 (33.3)	5 (12.8)				
Adjuvant trastuzumab								
No	40 (44.9)	4 (33.3)	9 (75.0)	20 (51.3)	0.414 / 0.366^*2^	0.207 / 0.104^*2^^*b^	0.420 / 0.212^*2^^*b^	0.413 / 0.313^*2^
Yes	42 (47.2)	8 (66.7)	3 (25.0)	17 (43.6)				
Unknown	7 (7.9)	0 (0.0)	0 (0.0)	0 (0.0)				
Adjuvant chemotherapy								
No	21 (23.6)	1 (8.3)	3 (25.0)	8 (20.5)	0.271 / 0.291^*2^	0.587 / 1.000^*2^	0.448 / 0.412^*2^	0.744 / 1.000^*2^
Yes	57 (64.0)	11 (91.7)	9 (75.0)	27 (69.2)				
Unknown	11 (12.4)	0 (0.0)	0 (0.0)	4 (10.3)				
Adjuvant hormone therapy								
No	7 (7.9)	9 (75.0)	12 (100.0)	37 (94.4)	0.031 / 0.027^*2^	<0.001 / <0.001^*2^^*b^	0.078 / 0.078^*2^	1.000 / 1.000^*2^
Yes	71 (79.8)	3 (25.0)	0 (0.0)	2 (5.1)				
Unknown	11 (12.4)	0 (0.0)	0 (0.0)	0 (0.0)				
First clinically relevant event								
Local recurrence	1 (1.1)	1 (8.3)	1 (8.3)	1 (2.6)	0.539 / 0.451^*2^	<0.001 / <0.001^*2^^*b^	0.800 / 0.911^*2^	0.012 / 0.008^*2^
Distant metastasis	11 (12.4)	2 (16.7)	5 (41.7)	7 (18.4)				
Contralateral breast cancer	0 (0.0)	0 (0.0)	2 (16.7)	0 (0.0)				
Second primary tumor	5 (5.6)	0 (0.0)	1 (8.3)	2 (5.3)				
Not occurred	58 (65.2)	7 (58.3)	2 (16.7)	24 (63.2)				
Unknown	8 (9.0)	1 (8.3)	0 (0.0)	1 (2.6)				
Death from other causes	6 (6.7)	1 (8.3)	1 (8.3)	3 (7.9)				
First metastases site								
Not occured	79 (88.8)	9 (75.0)	8 (66.7)	33 (84.6)	0.155	0.073	0.566	0.231
Visceral	3 (3.4)	0 (0.0)	2 (16.7)	1 (2.6)				
No visceral	4 (4.5)	1 (8.3)	1 (8.3)	3 (7.7)				
Both	3 (3.4)	2 (16.7)	1 (8.3)	2 (5.1)				

*2 excludes unknown category

*1 Parametric test: Independent two-sample Student's *t* test

*a Fisher's exact test

*b Chi-square test

### Overall survival (OS)

**Figure 2:**
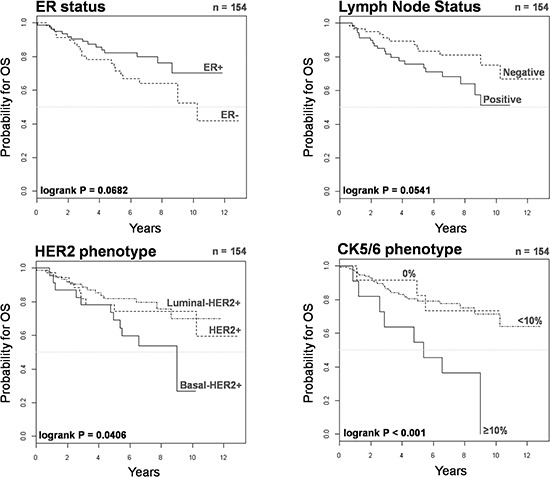
Kaplan-Meier OS curves of HER2+ patients stratified by ER status, lymph node status, HER2 phenotype, and CK5/6 phenotype

The 5-year Kaplan-Meier estimate of OS was 69% for patients with basal-HER2+ tumors, as compared with 82% for patients with luminal-HER2+ and 78% for HER2 + tumors (Figure 2). No statistical differences were found in 5-year estimated OS between patients with luminal-HER2+ tumors and those with HER2+ tumors. Upon stratification of basal-HER2+ to underlying CK5/6 expression, the 5-year Kaplan-Meier estimate of OS was 54% for patients with basal 2-HER2+ tumors (Figure 2), whereas the estimate of OS in patients with basal 1-HER2+ tumors (82%) was not statistically different to CK-negative (luminal-HER2+ and HER2+) HER2+ patients.

We utilized Cox's proportional-hazards regression model to assess OS (Table 3). Univariate analysis revealed that the presence of the basal-like phenotype was a significant predictor of a worse 5-year OS (hazard ratio 2.36, 95% confidence interval 1.18–4.75; *P* = 0.0159). Upon stratification of basal CK5/6 expression pattern, univariate analysis revealed that a positive CK5/6 staining in ≥ 10% of the HER2+ breast cancer tissue sections (but not a positive CK5/6 < 10%) predicted a significantly worse prognosis in terms of 5-year OS (hazard ratio 4.07, 95% confidence interval 1.88–8.79; *P* < 0.001). Neither ER nor nodal statuses were statistically significant prognostic factors of 5-year OS. When variables correlating with univariate survival at *p* < 0.20 were later included in multivariate Cox regression analysis (Table 3), the basal-like phenotype lost its independent value for predicting OS. In multivariate analysis, only age at diagnosis and tumor size remained independent predictors of OS.

**Table 3:** Cox regression analysis of factors predicting OS

Characteristics	Univariate analysis		Multivariate analysis ^*1^	
	Hazard ratio (95% confidence interval)	*P*	Hazard ratio (95% confidence interval)	*P*
Age (continuous)	1.07 (1.04–1.10)	<0.001	1.06 (1.03–1.09)	<0.001
Tumor size				
T1	1		1	
T2	2.24 (0.81–6.25)	0.122	1.87 (0.66–5.32)	0.239
T3+T4	7.84 (2.68–22.88)	<0.001	5.75 (1.92–17.24)	0.002
Inflammatory	5.09 (1.19–21.69)	0.028	5.51 (1.29–23.60)	0.022
Lymph Node status				
Negative	1			
Positive	2.01 (0.97−4.14)	0.059		
Tumor grade				
1+2	1			
3	1.61 (0.68–3.81)	0.279		
ER status				
Negative	1			
Positive	0.54 (0.28–1.06)	0.072		
HER2+ phenotype				
Luminal-HER2+	1			
HER2+	1.25 (0.54–2.86)	0.600		
Basal-HER2+	2.55 (1.19–5.46)	0.016		
Basal phenotype				
Absent	1			
Present	2.36 (1.18–4.75)	0.0159		
CK5/6 phenotype				
0%	1			
< 10%	1.05 (0.31–3.49)	0.942		
≥ 10%	4.07 (1.88–8.79)	<0.001		
HER2+ Subtype				
Luminal-HER2+	1			
HER2+	1.25 (0.54–2.86)	0.599		
Basal 1-HER2+ (< 10%)	1.13 (0.33–3.91)	0.849		
Basal 2-HER2+ (≥ 10%)	4.39 (1.91–10.05)	<0.001		
Adjuvant trastuzumab				
No	1			
Yes	0.87 (0.43–1.80)	0.714		
Adjuvant chemotherapy				
No	1			
Yes	0.82 (0.38–1.78)	0.615		
Adjuvant hormone therapy				
No	1			
Yes	0.37 (0.18–0.77)	0.007		

*1 proportional hazard assumption for the Cox model has been checked

### Disease-free survival (DFS)

**Figure 3:**
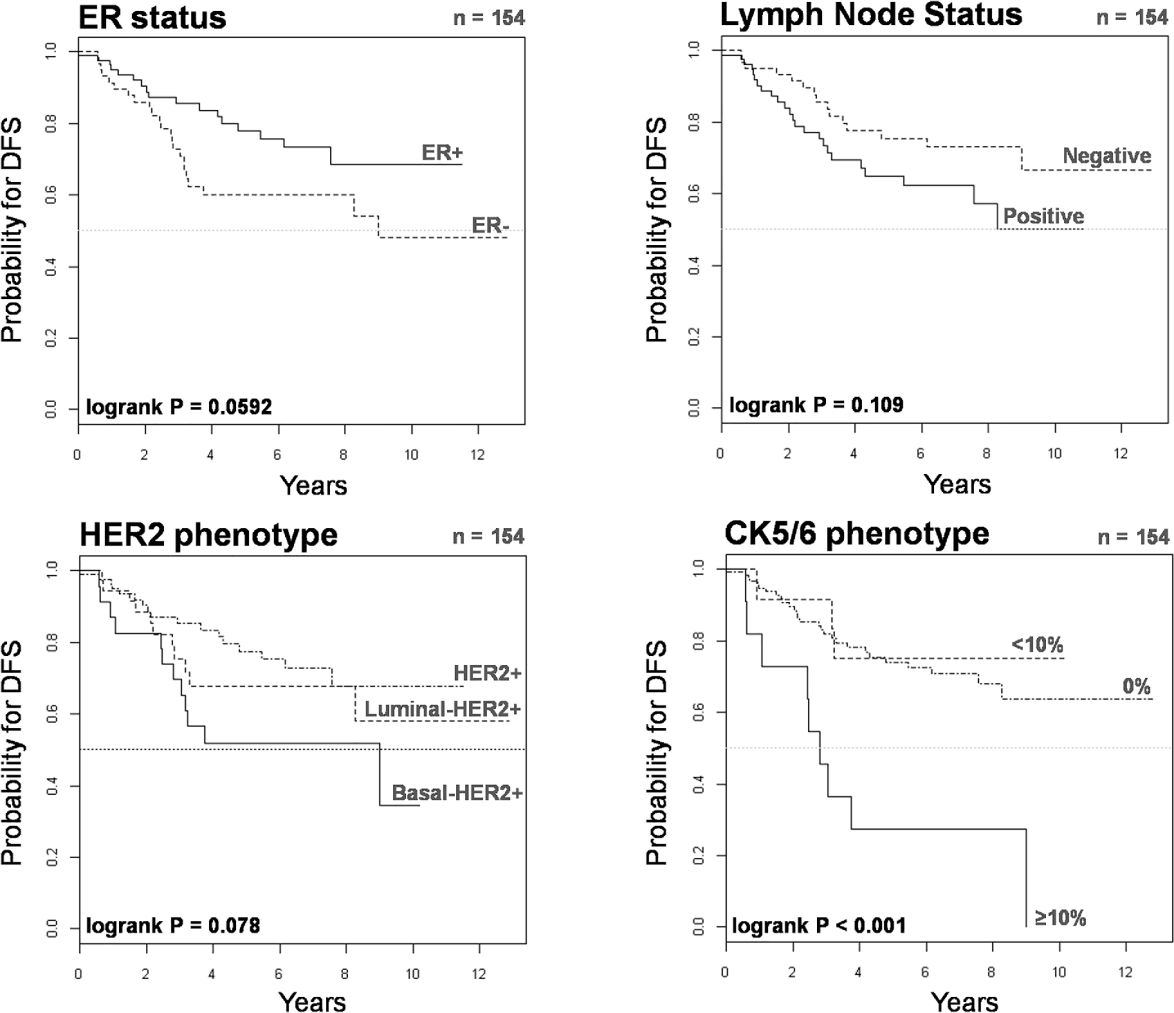
Kaplan-Meier DFS curves of HER2+ patients stratified by ER status, lymph node status, HER2 phenotype, and CK5/6 phenotype

The 5-year Kaplan-Meier estimate of DFS was 52% for patients with basal-HER2+ tumors, as compared with 77% for patients with luminal-HER2+, and 68% for HER2 + tumors (Figure 3). No statistical differences were found in 5-year estimated DFS between patients with luminal-HER2+ tumors and patients with HER2+. Notably, upon stratification of basal-HER2+ to underlying CK5/6 expression pattern, the 5-year Kaplan-Meier estimate of DFS was as low as 27% for patients with basal 2-HER2+ tumors (Figure 3), whereas the estimate of DFS in patients with basal 1-HER2+ tumors (75%) was not statistically different to those with basal CK-negative (luminal-HER2+ and HER2+) HER2+ patients.

We utilized Cox's proportional-hazards regression model to assess DFS (Table 4). Univariate analysis revealed that the presence of the basal-like phenotype was a significant predictor of a worse 5-year DFS (hazard ratio 2.05, 95% confidence interval 1.04–4.04; *P* = 0.037). Upon stratification of basal CK5/6 expression pattern, univariate analysis revealed that a positive CK5/6 staining in ≥ 10% of the HER2+ breast cancer tissue sections (but not a positive CK5/6 < 10%) predicted a significantly worse 5-year DFS (hazard ratio 4.17, 95% confidence interval 1.96–8.98; *P* < 0.001). Neither ER nor nodal statuses were statistically significant prognostic factors of 5-year DFS. Importantly, when variables correlating with univariate survival at *p* < 0.20 were later included in multivariate Cox regression analysis (Table 4), the presence of the basal 2-HER2+ phenotype (i.e., a positive CK5/6 staining in more than 10% of the HER2+ breast cancer tissue sections) retained its independent value for predicting a worse outcome in terms of DFS (hazard ratio 2.44, 95% confidence interval 1.05–5.67; *P* = 0.037). In multivariate analysis, tumor size also remained an independent predictor of a worse DFS.

**Table 4:** Cox regression analysis of factors predicting DFS

Characteristics	Univariate analysis		Multivariate analysis ^*1^	
	Hazard ratio (95% confidence interval)	*P*	Hazard ratio (95% confidence interval)	*P*
Age (continuous)	1.02 (1.00–1.04)	0.115		
Tumor size				
T1	1		1	
T2	3.38 (1.27–9.02)	0.015	3.05 (1.13–8.23)	0.028
T3+T4	7.74 (2.64–22.71)	<0.001	6.41 (2.05–20.05)	0.001
Inflammatory	5.21 (1.23–22.00)	0.025	5.17 (1.21–21.98)	0.026
Node status				
Negative	1			
Positive	1.69 (0.88–3.23)	0.113		
Tumor grade				
1+2	1			
3	1.01 (0.48–2.10)	0.989		
ER status				
Negative	1			
Positive	0.55 (0.29–1.03)	0.063		
HER2+ phenotype				
Luminal-HER2+	1			
HER2+	1.37 (0.64–2.93)	0.416		
Basal-HER2+	2.29 (1.10–4.82)	0.028		
Basal phenotype				
Absent	1			
Present	2.05 (1.04–4.04)	0.037		
CK5/6 phenotype				
0%	1	1		
< 10%	0.81 (0.25–2.68)	0.734	0.60 (0.18–2.02)	0.409
≥ 10%	4.17 (1.96–8.87)	<0.001	2.44 (1.05–5.67)	0.037
HER2+ Subtype				
Luminal-HER2+	1			
HER2+	1.37 (0.64–2.93)	0.415		
Basal 1-HER2+ (< 10%)	0.91 (0.27–3.11)	0.880		
Basal 2-HER2+ (≥ 10%)	4.67 (2.08–10.51)	<0.001		
Adjuvant trastuzumab				
No	1			
Yes	0.98 (0.51–1.87)	0.953		
Adjuvant chemotherapy				
No	1			
Yes	0.82 (0.40–1.70)	0.590		
Adjuvant hormone therapy				
No	1			
Yes	0.60 (0.32–1.13)	0.111		

*1 proportional hazard assumption for the Cox model has been checked

### Time to treatment failure (TTF)

**Figure 4:**
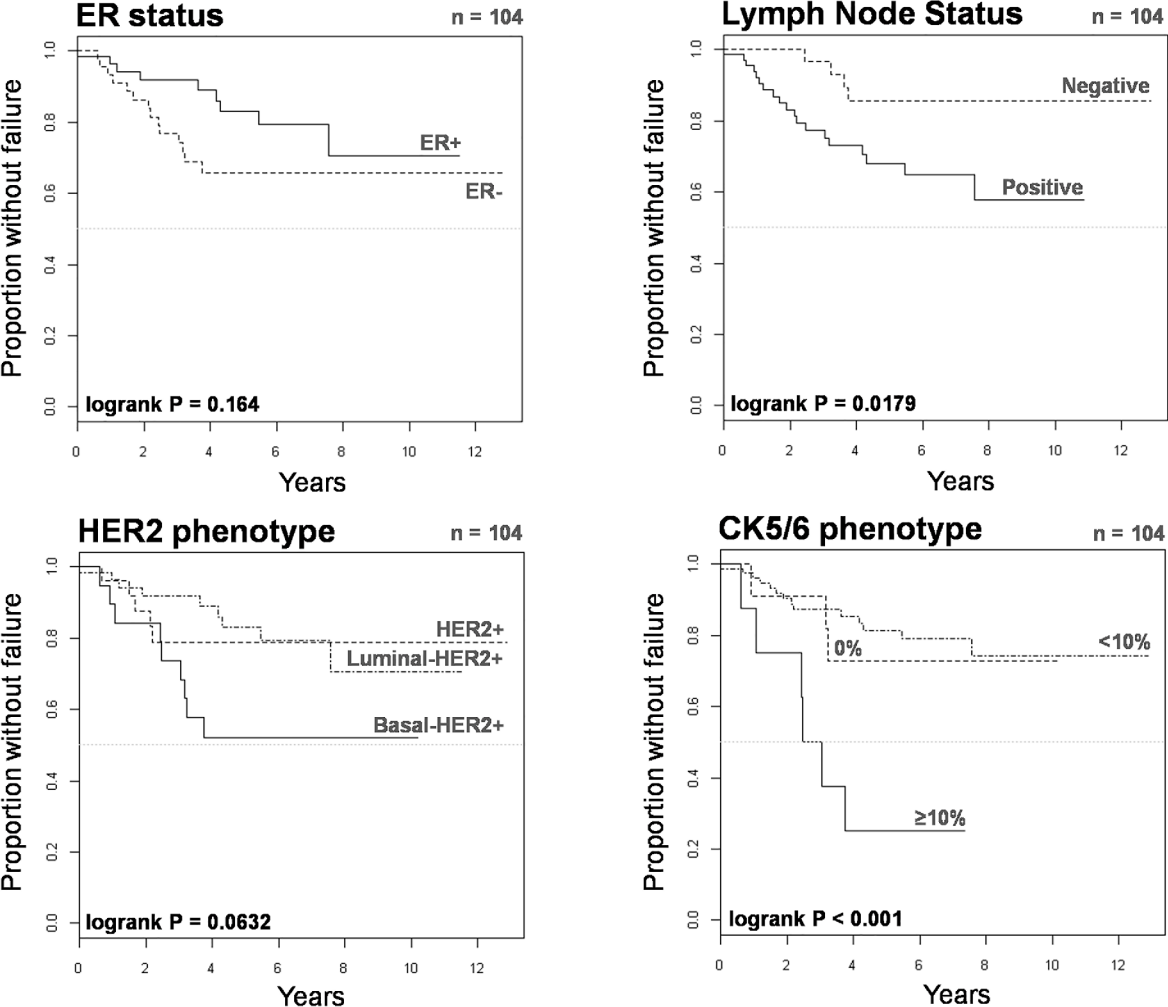
Kaplan-Meier TTF curves of HER2+ patients treated with chemotherapy only or trastuzumab-based therapy when stratified by ER status, lymph node status, HER2 phenotype, and CK5/6 phenotype

We investigated the prognostic significance of the basal-HER2+ phenotype in 104 HER2+ patients treated with either chemotherapy-only or trastuzumab-based adjuvant (*n* = 67, 64%)/neoadjuvant (*n* = 37, 36%) chemotherapy. We utilized Cox's proportional-hazards regression model to assess whether the basal-HER2+ might constitute a predictor of a worse course in terms of time to treatment failure (TTF) (Table 5). Univariate analysis revealed that tumor size, node status and the presence of a basal-like phenotype predicted a significantly worsened TTF (Figure 4). Upon stratification of basal CK5/6 expression pattern, univariate analysis revealed that a positive CK5/6 staining in ≥ 10% of the HER2+ breast cancer tissue sections (but not a positive CK5/6 < 10%) was a significant predictor of worse outcome in terms of TTF (hazard ratio 5.45, 95% confidence interval 2.07–14.35; *P* < 0.001). Remarkably, after accounting for prognostic variables correlating with univariate survival at *p* < 0.20 (Table 5), tumor size and the presence of the basal 2-HER2+ phenotype (i.e., a positive CK5/6 staining in ≥ 10% of the HER2+ breast cancer tissue sections) retained their independent value for predicting a worse prognosis in terms of TTF (hazard ratio 3.66; 95% confidence interval 1.24–10.78; *P* = 0.019) in multivariate Cox regression analysis.

**Table 5:** Cox regression analysis of factors predicting TTF

Characteristics	Univariate analysis		Multivariate analysis ^*1^	
	Hazard ratio (95% confidence interval)	*P*	Hazard ratio (95% confidence interval)	*P*
Age (continuous)	1.01 (0.98–1.05)	0.384		
Tumor size				
T1	1		1	
T2	7.74 (1.01–59.55)	0.049	6.73 (0.87–52.25)	0.068
T3+T4	19.80 (2.38–165.03)	0.006	11.64 (1.29–104.96)	0.029
Inflammatory	16.03 (1.65–155.62)	0.017	15.09 (1.54–147.66)	0.020
Node status				
Negative	1			
Positive	3.42 (1.16–10.08)	0.026		
Tumor grade				
1+2	1			
3	0.90 (0.31–2.55)	0.836		
ER status				
Negative	1			
Positive	0.56 (0.24–1.29)	0.170		
HER2+ phenotype				
Luminal-HER2+	1			
HER2+	1.11 (0.37–3.33)	0.848		
Basal-HER2+	2.72 (1.08–6.87)	0.034		
Basal phenotype				
Absent	1			
Present	2.63 (1.14–6.07)	0.024		
CK5/6 phenotype				
0%	1		1	
< 10%	1.30 (0.37–4.52)	0.684	1.26 (0.36–4.47)	0.717
≥ 10%	5.45 (2.07–14.35)	<0.001	3.66 (1.24–10.78)	0.019
HER2+ Subtype				
Luminal-HER2+	1			
HER2+	1.11 (0.37–3.32)	0.855		
Basal 1-HER2+ (< 10%)	1.34 (0.36–4.97)	0.659		
Basal 2-HER2+ (≥ 10%)	5.64 (1.99–15.99)	0.001		
Treatment				
Only Chemotherapy	1			
Trastuzumab + Chemotherapy	0.99 (0.43–2.30)	0.989		

*1 proportional hazard assumption for the Cox model has been checked

**Figure 5:**
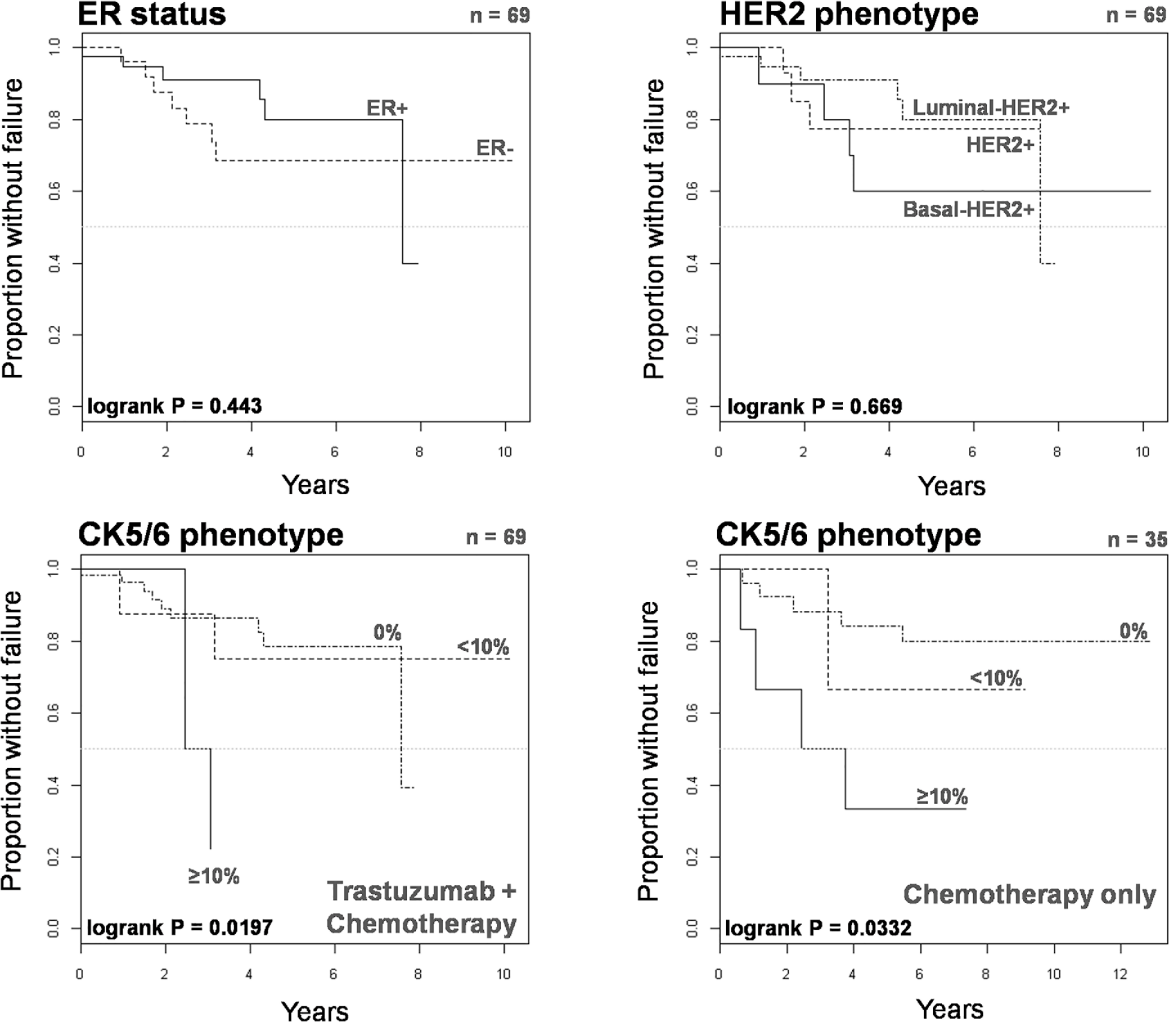
Kaplan-Meier TTF curves of HER2+ patients treated with trastuzumab-based therapy when stratified by ER status, HER2 phenotype, and CK5/6 phenotype

We finally assessed the prognostic significance of the basal-HER2+ phenotype in 69 HER2+ patients treated with trastuzumab-based adjuvant (*n* = 42, 61%)/neoadjuvant (*n* = 27, 39%) therapy (Table 6). Upon stratification of basal CK5/6 expression pattern, univariate analysis confirmed that a positive CK5/6 staining in ≥ 10% of the HER2+ breast cancer tissue sections (but not a positive CK5/6 < 10%) was a significant predictor of worse outcome in terms of TTF in trastuzumab-treated patients (hazard ratio 7.49, 95% confidence interval 1.41–39.70; *P* = 0.018). More importantly, a positive CK5/6 staining in ≥ 10% of the HER2+ breast cancer tissue sections remained the sole independent factor predicting a worse outcome of trastuzumab-treated patients in terms of TTF in multivariate Cox regression analysis (hazard ratio 6.80, 95% confidence interval 1.39–33.36). Kaplan-Meier estimates of TTF confirmed that patients with basal 2-HER2+ tumors did not benefit from adding trastuzumab to chemotherapy (Figure 5).

**Table 6:** Cox regression analysis of factors predicting time to trastuzumab failure

Characteristics	Univariate analysis		Multivariate analysis ^*1^	
	Hazard ratio (95% confidence interval)	*P*	Hazard ratio (95% confidence interval)	*P*
Age (continuous)	1.03 (0.98–1.08)	0.264		
Tumor size				
T1	1			
T2	4.72 (0.57–39.34)	0.152		
T3+T4	14.19 (1.43–140.77)	0.024		
Inflammatory	7.05 (0.63–78.70)	0.113		
Node status				
Negative	–	–		
Positive	–	–		
Tumor grade				
1+2	1			
3	1.27 (0.33–4.92)	0.731		
ER status				
Negative	1			
Positive	0.65 (0.22–1.95)	0.447		
HER2+ phenotype				
Luminal–HER2+	1			
HER2+	1.29 (0.32–5.18)	0.724		
Basal–HER2+	1.79 (0.49–6.46)	0.376		
Basal phenotype				
Absent	1			
Present	1.66 (0.50–5.54)	0.408		
CK5/6 phenotype				
0%	1		1	
< 10%	0.89 (0.18–4.43)	0.888	0.89 (0.18–4.43)	0.888
≥ 10%	6.80 (1.39 –33.36)	0.018	6.80 (1.39 –33.36)	0.018
HER2+ Subtype				
Luminal–HER2+	1			
HER2+	1.34 (0.33–5.43)	0.684		
Basal 1–HER2+ (< 10%)	0.96 (0.19–4.97)	0.966		
Basal 2–HER2+ (≥ 10%)	7.49 (1.41–39.70)	0.018		

*1 proportional hazard assumption for the Cox model has been checked

## DISCUSSION

Currently available gene expression signatures cannot identify HER2+ subgroups of good- and poor-responders to trastuzumab. In an ambitious attempt to address the need for a predictor of trastuzumab benefit, Pogue-Geile *et al*. [8] developed an eight-gene model able to stratify HER2+ tumors into a discontinuous distribution of patients within 3 different subsets of “extraordinary”, “moderate”, and “no apparent” benefit from trastuzumab. This complexity makes the establishment of optimal cutoffs nearly impossible and the validation of the eight-gene classifier, if applied arbitrarily, could lead to misclassification of HER2+ patients and thus inappropriate treatment recommendations [22]. Using molecular profiling to investigate the variable prognosis and response to therapy of HER2+ tumors, Staaf *et al*. [23] identified a 158-gene HER2-derived prognostic predictor (HDPP) that improved the stratification of good and poor prognosis for both OS and distant metastasis-free survival in both the HER2-enriched molecular subtype and the basal-like subtype, which are assumed to be mutually exclusive breast cancer entities. When applied to a small HER2+ group of patients preoperatively treated with trastuzumab, the HDPP signature correlated with the occurrence of trastuzumab resistance. Although the sample size was insufficient to form any conclusion, it should be noted that when formerly analyzing this data set, Harris *et al*. [9] posited that “*HER2-overexpressing tumors with a basal-like phenotype*” were more likely to be intrinsically resistant to pre-operative trastuzumab. Here, we present evidence that immunohistochemical reclassification of HER2+ breast cancer tumors by basal CK5/6 expression might be sufficient to significantly improve prognosis and trastuzumab-based therapeutic planning.

Previous morpho-immunohistologic studies [15–19] have shown that a subgroup of HER2+ tumors consistently demonstrate several features classically ascribed to basal-like tumors, including poor differentiation, high-grade, geographic necrosis, pushing margins of invasion, syncytial arrangement of tumor cells, ribbon- or festoon-like, squamous metaplasia, stromal lymphocytic infiltrates, high mitotic index, and p53 positivity. Importantly, these basal-HER2+ breast carcinomas show immunoreactivity for the basal epithelium cytokeratin marker CK5/6, which at times appears as a uniform or almost uniform positive staining that fully mimics the pattern of pure basal-like breast carcinomas, but often displays checkerboard-type intratumoral heterogeneity. Here we confirm that the basal-HER2+ phenotype defined by the immunohistochemical expression of basal CK5/6 is associated with aggressive disease and adversely impacts survival in HER2+ breast cancer patients. In our series, there were no statistically significant differences in OS and DFS between patients with ER-positive luminal-HER2+ tumors and ER-negative HER2+ tumors. However, the basal-HER2+ phenotype was significantly associated with inferior OS by univariate analysis and, after accounting for strong prognostic variables such as tumor size at diagnosis in stepwise multivariate analysis, the presence of the basal-HER2+ phenotype, but not ER status, also predicted significantly worsened DFS when a positive CK5/6 staining in ≥ 10% of the HER2+ breast cancer tissue sections was registered as a diagnostically relevant positive reaction.

Our findings might not only have important implications for prognosis, but also for therapy. The simple and specific CK5/6 fingerprint using a 10% cutoff allows the re-classification of HER2+ tumors into a sub-class of basal 1-HER2+ tumors (<10% of cells showing positive CK5/6 staining), which appear to be prognostically indistinguishable from HER2+ tumors, and a sub-class of clinically aggressive basal 2-HER2+ tumors (≥10% of cells showing positive CK5/6 staining) which will likely be unresponsive to trastuzumab-based adjuvant/neoadjuvant therapy. While acknowledging that our study is small and exploratory and the data on adjuvant/neoadjuvant trastuzumab are limited in size (approximately 45% of HER2+ patients) and maturity (median follow-up is approximately 4 years for patients treated with adjuvant trastuzumab), the fact that the use of adjuvant/neoadjuvant trastuzumab and chemotherapy did not greatly differ between patients with HER2+ and basal 2-HER2+ tumors together with the strong association between the basal 2-HER2+ phenotype and worsened survival and shorter time to trastuzumab-based treatment failure, strongly suggest that immunohistochemical identification of the basal 2-HER2+ phenotype may be used as a predictive marker of primary refractoriness to trastuzumab. Accordingly, the co-expression of well-known basal-like molecular features, including expression of the anti-apoptotic protein survivin, the dynamic emergence of the CD44^+^CD24^−/low^ breast cancer stem cell (CSC) immunophenotype, or the occurrence of epithelial-to-mesenchymal transition (EMT) phenomena, occurs exclusively in HER2+ breast cancer cells exhibiting primary resistance to trastuzumab [10, 19, 24–30].

**Figure 6:**
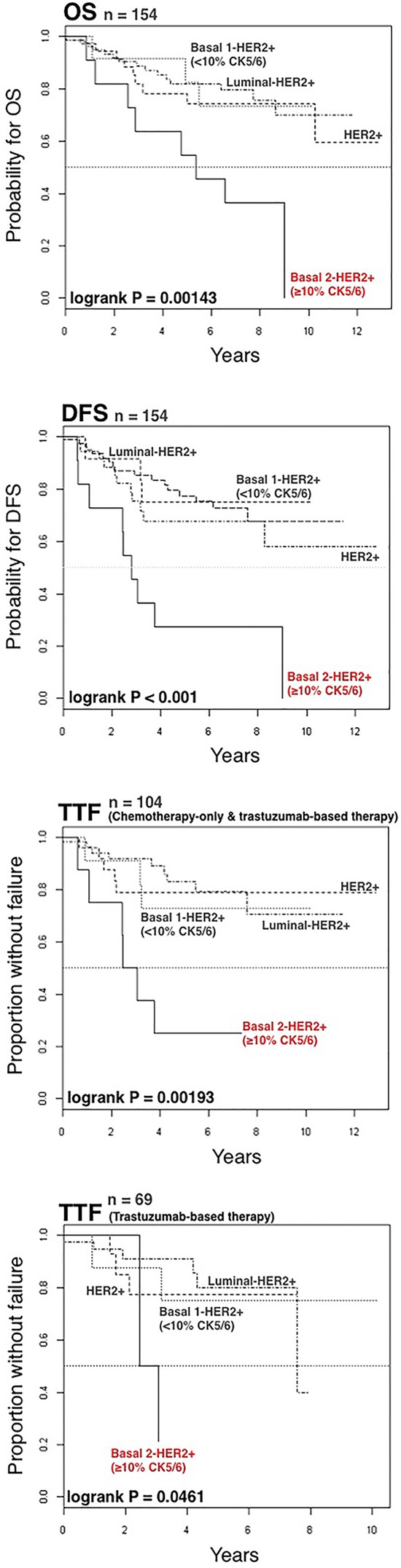
Kaplan-Meier OS, DFS, and TTF curves for patients with luminal-HER2+, HER2+, basal 1-HER2+ (<10% CK5/6), and basal 2-HER2+ (≥10% CK5/6) tumors as defined by immunohistochemical analysis (Figure 1)

**Figure 7:**
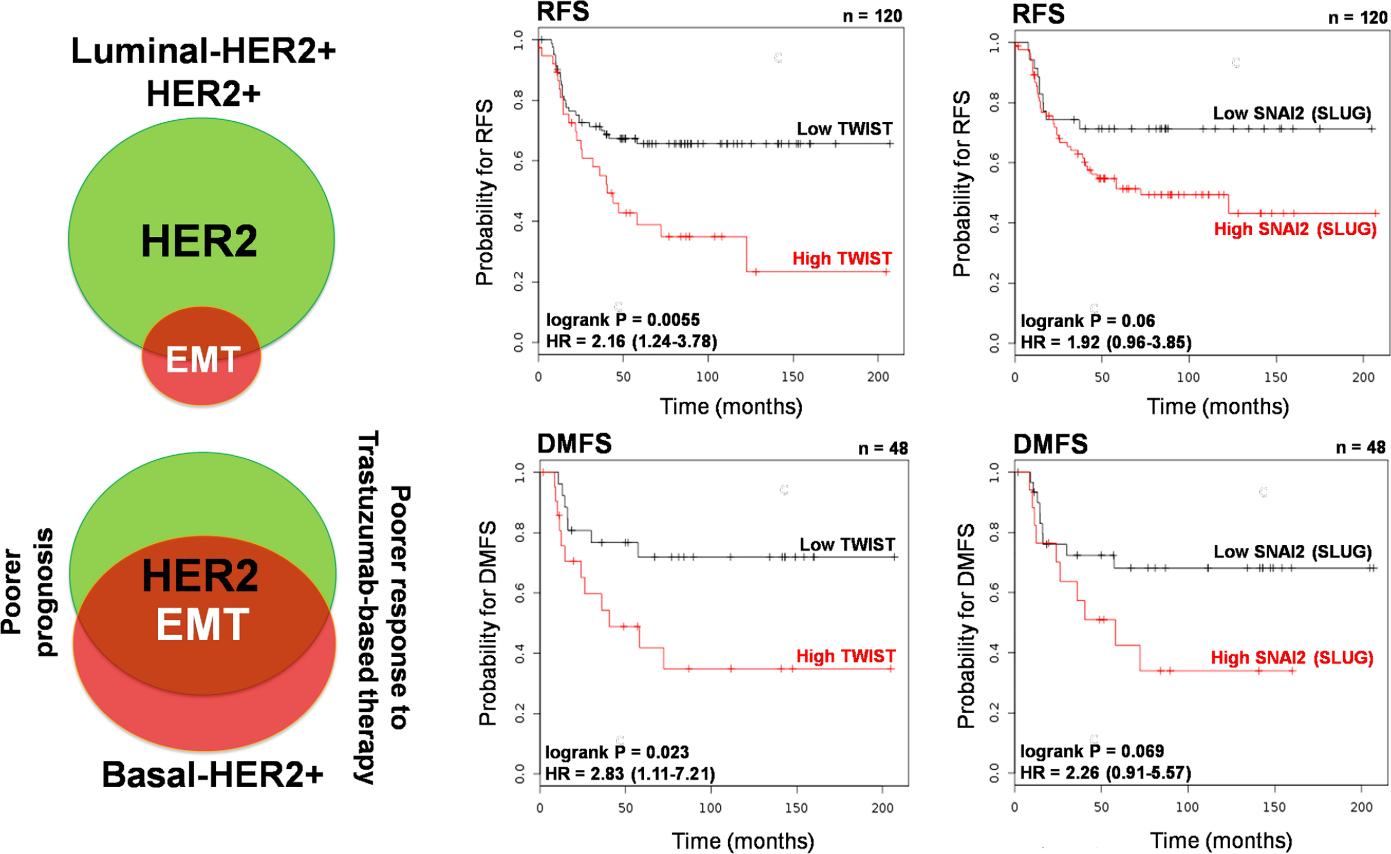
Kaplan-Meier RFS and DMFS curves of HER2+ patients stratified by low/high TWIST and low/high SNAI2 (SLUG) gene expression

The *a priori* characterization of distinct biological HER2+ breast cancer subgroups associated with poorer prognosis and resistance to trastuzumab-based adjuvant/neoadjuvant therapy using DNA microarrays is not currently feasible for large-scale clinical applications. In this setting, immunohistochemical staining of basal CK5/6 can be a useful surrogate to predict inferior survival and poorer responses to trastuzumab-based therapy. In our hands, a CK5/6-based fingerprint using a 10% positivity cutoff allows the rapid re-classification of HER2+ tumors in a manner that improves prognosis and therapeutic planning in a sub-class of patients with clinically aggressive basal-HER2+ tumors who are unlikely to benefit from trastuzumab-based adjuvant therapy (Figure 6). It is tempting to suggest that CK5/6-defined basal-HER2+ tumors might be viewed as an immunohistochemical algorithm, analogous to EMT and CSC-like gene signatures and likely contributes to the poor outcomes in basal-HER2+ tumors [31–36]. Although larger retrospective studies should be conducted to unequivocally determine whether basal-HER2+ tumors are differentially enriched with cells combining EMT/CSC phenotypes, it is noteworthy that the sole re-classification of ER-/HER2+ tumors by the expression of the core EMT transcription factors SNAI2 (SLUG) and TWIST [37] using the on-line Kaplan-Meier plotter (http://kmplot.com/) [38, 39], was sufficient to predict a significantly inferior relapse free survival (RFS) and distant metastasis free survival (DMFS) in the EMT-like/HER2+ patient cohort (Figure 7).

There is an urgent need to generate a more definitive taxonomy of HER2+ breast carcinomas as well as molecular forecasting signatures to be validated prospectively and in samples from trastuzumab-based clinical trials. Our current findings lend support to forthcoming prospective studies aimed to validate the concept that identification of CK5/6-positive immunophenotypes within HER2+ breast carcinomas may be a rapid and accurate method for identifying intrinsic subgroups of biologically aggressive tumors likely to display resistance to trastuzumab *ab initio* in women diagnosed with HER2+ cancer.

## METHODS

### Study patients

The prospectively maintained breast cancer database at the Hospital Universitari de Girona Dr. Josep Trueta in Girona, Spain was queried to identify patients diagnosed with HER2-overexpressing primary breast cancer between June 2000 and February 2014. The study was limited to the most common breast cancer histology, i.e., invasive ductal cancer, to allow for more accurate prognostication. The investigators irreversibly anonymized (stripped of any link to the original patient) all the tissues and existing data in such a manner that subjects cannot be identified, directly or through identifiers linked to the subjects.

### Immunohistochemistry (IHC) and classification

Primary tumor size according to pathological analysis was classified according to the Seventh Edition of the AJCC Cancer Staging Manual. A board-certified specialty-trained breast pathologist (E. L. B.) reviewed immunohistochemical (ER, HER2) stainings from 154 consecutive patients with HER2-overexpressing primary invasive ductal breast cancer. Approximately 30% of the study cohort was previously checked for receptor expression, which was undertaken as an internal quality control measure to confirm that the ER and HER2 status of patients at the time of performance of this study was in agreement with that initially rendered at the time of diagnosis. No noticeable differences were encountered when ER and HER2 IHC staining was scored using criteria from published guidelines. The cutoff for ER immunoreactivity was 10% positive tumor nuclei irrespective of intensity. HER2 expression status was considered positive if immunostaining was scored as 3+ (i.e., strongly positive in > 10% of cancer cells by IHC) according to HercepTest criteria. For an equivocal result of HER2 2+ (i.e., moderately positive in > 10% of cancer cells by IHC), HER2 expression status was considered positive if fluorescent *in situ* hybridization (FISH) assay revealed a HER2:chromosome-17 amplification ratio of > 2.2.

Paraffin-embedded tissue sections from HER2+ tumors were immunostained with CK5/6 (clone D5/16B4, Dako; 1:50 dilution) antibody. Antigen retrieval was performed using Tris-EDTA pH 9 for 30 minutes. Analysis was carried out by a board-certified pathologist (E. L. B.), who scored the basal CK5/6 staining on a scale of 0–2: 0, no staining; 1, < 10% of cells showing positive staining; and 2, ≥ 10% of cells showing positive. A CK5/6 score greater than 0 originally defined a positive basal phenotype. Tumors were then classified as luminal/HER2+ subtype (ER positive and basal CK5/6 negative), HER2+ subtype (ER negative and basal CK5/6 negative), and basal-HER2+ subtype (ER negative and basal CK5/6 positive) (Figure 1).

The current study is reported according to the Reporting Recommendations for Tumor Marker Prognostic Studies (REMARK). Laboratory personnel who were blinded to clinical data and outcomes performed all IHC assays. Assay results were interpreted and scored by a pathologist (E. L. B.) who remained blinded to clinical data.

### Statistical analysis

All statistical data analyses were performed using IBM SPSS version 21.0 (IBM Corp. 2012) and R software environment (http://www.r-project.org/). Data collected included date of birth, date of diagnosis, tumor size, tumor grade, lymph node status, receipt of adjuvant therapy (hormone, trastuzumab, and chemotherapy), site and date of recurrent disease, and date of death. Proportions observed among categorical variables were compared using the chi-squared test or Fisher's exact test. The Mann-Whitney *U* test was used to test for differences in continuous variables. Survival functions were estimated for disease-free survival and overall survival using the product-limit method of Kaplan and Meier. Overall survival (OS) was calculated from the date of diagnosis to the date of death. Disease-free survival (DFS) was calculated from the date of initial diagnosis to the date of recurrence or death, whichever came first. Patients not experiencing an event were considered censored at the date of last contact. Time to treatment failure (TTF) was calculated from the date of therapy initiation to the time of first evidence of failure, i.e., disease progression, recurrence or death. Inference on survival functions among subgroups was based on the log-rank test for the equality of the survival functions. Cox's proportional-hazards regression model was used to identify statistically significant predictors of OS, DFS, and TTF and the proportional hazard assumption was validated. In such models, the covariate function is proportional to the hazard or mortality and, therefore, positive coefficients indicate a shorter survival with increasing value of the covariate. *P* values of less than 0.05 were considered to be statistically significant.
